# Temporoparietal Scalp Intravenous Schwannoma: Rare Subcutaneous Soft Tissue Mass in an Unusual Location

**DOI:** 10.1155/cris/8389899

**Published:** 2026-09-15

**Authors:** Maedot A. Haymete, Austin L. Fitzgerald, Jane J. Gay, Daniel R. Tershak, Kristin B. Eden, Douglas J. Grider

**Affiliations:** ^1^ Virginia Tech Carilion School of Medicine, Roanoke, Virginia, USA, vt.edu; ^2^ Division of Dermatology, Department of Internal Medicine, Virginia Tech Carilion School of Medicine, Roanoke, Virginia, USA, vt.edu; ^3^ Department of Surgery, Virginia Tech Carilion School of Medicine, Roanoke, Virginia, USA, vt.edu; ^4^ Department of Basic Science Education, Virginia Tech Carilion School of Medicine, Roanoke, Virginia, USA, vt.edu; ^5^ Dominion Pathology Associates, Roanoke, Virginia, USA

## Abstract

Schwannomas, or neurilemmomas, are benign nerve sheath tumors of Schwann cell origin, sometimes associated with neurofibromatosis type 2 (NF2) and schwannomatosis. Intravenous schwannomas are exceedingly rare, pose diagnostic challenges due to their unusual location within blood vessels and lack of surrounding perineurium. A 69‐year‐old male presented with a subcutaneous mass behind the left ear in the upper temporoparietal scalp, firm and mobile, 5 × 7 cm^2^, gradually growing for 15 years. Past physical examinations considered the lesion a cutaneous inclusion cyst. Surgical extirpation revealed a solid tumor adherent to the underlying fascia and muscle easily removed along surgical planes. Histopathology showed a well‐circumscribed, spindle cell lesion, S100 protein positive, with foci of hypercellular (Antoni A) and hypocellular (Antoni B) areas, with distinctive Verocay bodies pathognomonic for schwannoma. EMA was negative at the periphery, demonstrating a lack of perineurium, supporting that the schwannoma was not within a nerve bundle. However, a Verhoeff van Gieson elastic tissue stain revealed a circumferential single internal elastic lamina, while CD31 marked the endothelial cells lining a stretched vein encasing the tumor, confirming an intravenous schwannoma.

## 1. Introduction

Schwannomas, also called neurilemmomas, are benign nerve sheath tumors that originate from Schwann cells and have been found to be associated with neurofibromatosis type 2 (NF2) and schwannomatosis, though this is not always the case [1, 2]. They most commonly present as solitary, encapsulated masses along peripheral nerves within the deep dermis or subcutis. [3] Histologically, they are characterized by spindle cell proliferation with alternating Antoni A and B areas and may demonstrate Verocay bodies, which are pathognomonic for schwannomas [3, 4]. Schwannomas are typically slow‐growing and may be asymptomatic or cause localized discomfort due to the compression of adjacent structures [4]. Additionally, the size and growth patterns of schwannomas can also vary widely, posing diagnostic challenges in clinical practice [5].

In contrast to conventional schwannomas, intravascular schwannomas, venous or arterial, are exceptionally rare, with only four prior cases reported in the literature. Reported cases have primarily involved small‐ to medium‐sized vessels, most commonly in the extremities or central vasculature. Because of their unusual location, these lesions are frequently not suspected clinically and may be misdiagnosed as more common subcutaneous masses, such as hair follicle inclusion cysts [1, 6–8].

Presented is a case of intravenous schwannoma arising in the subcutaneous soft tissue of the temporoparietal scalp, an anatomic location that has not previously been reported for intravascular schwannoma. This case highlights the diagnostic challenges associated with this rare entity and expands the current understanding of its clinical presentation and distribution.

## 2. Case Report

A 69‐year‐old male presented to the surgery clinic for the evaluation of a subcutaneous mass behind his left ear. He reported a gradual, progressive growth of the lesion since he first noticed it 15 years ago. He also mentioned that the mass seemed to fluctuate in size. There was no history of drainage. The lesion was asymptomatic other than pain at times when trying to sleep. A complete review of symptoms was otherwise negative.

**Figure 1:**
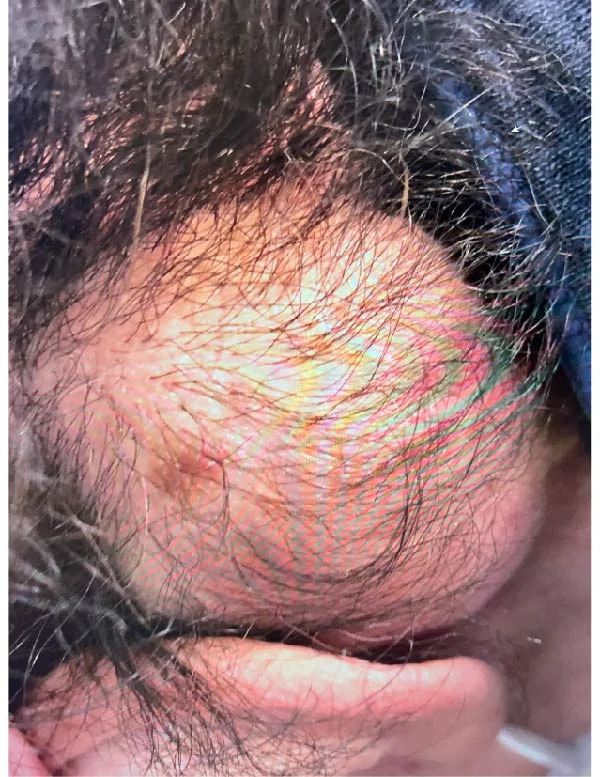
Clinical photograph of the large, flesh‐colored mass behind the left posterior ear.

Physical examination revealed a large, firm, and mobile mass measuring 7 × 5 cm^2^, posterior to the left earlobe (Figure 1). Given the clinical history of a mass slowly growing over 15 years and the physical findings noted, a cutaneous inclusion cyst, such as an epidermal inclusion cyst (EIC) or pilar cyst, was the favored presurgical diagnosis. Since preoperative imaging is not standard prior to surgical intervention for a cutaneous inclusion cyst, imaging was not performed prior to surgical extirpation.

**Figure 2:**
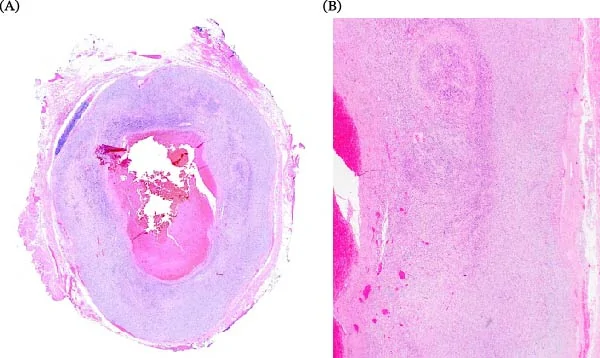
(A) H&E‐stained tissue section of the spindle cell lesion within a blood vessel (whole slide scanned image). (B) H&E‐stained tissue section of the spindle cell lesion with Antoni A and B areas (2x).

**Figure 3:**
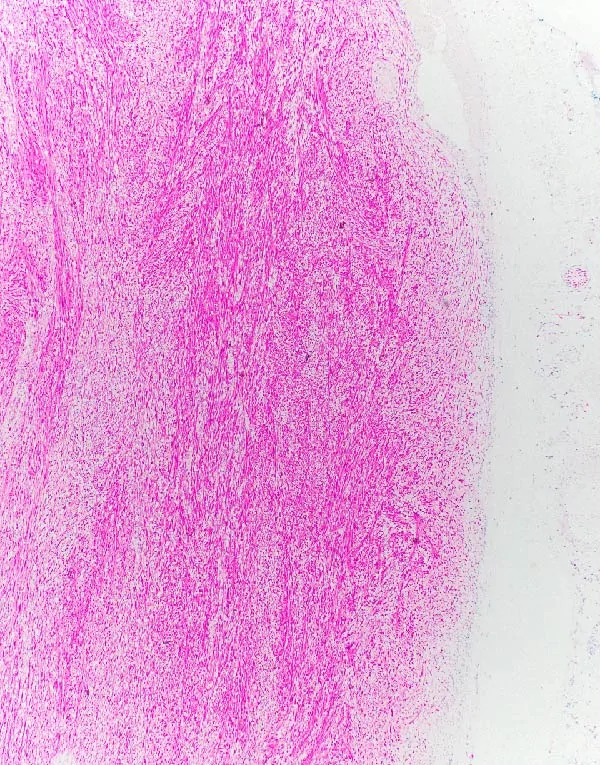
S100 protein immunohistochemically stained tissue section marking the spindle cell proliferation (4x).

**Figure 4:**
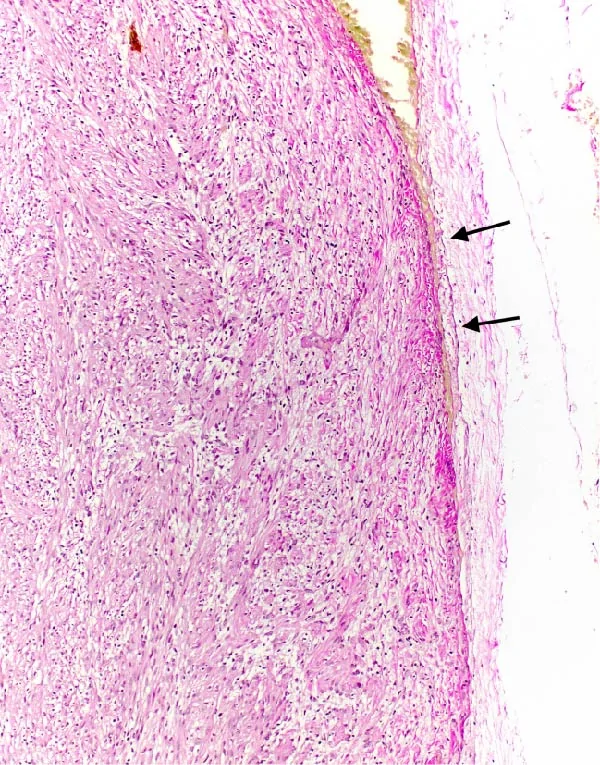
Verhoeff‐Van Gieson elastic stained tissue section (10x).

**Figure 5:**
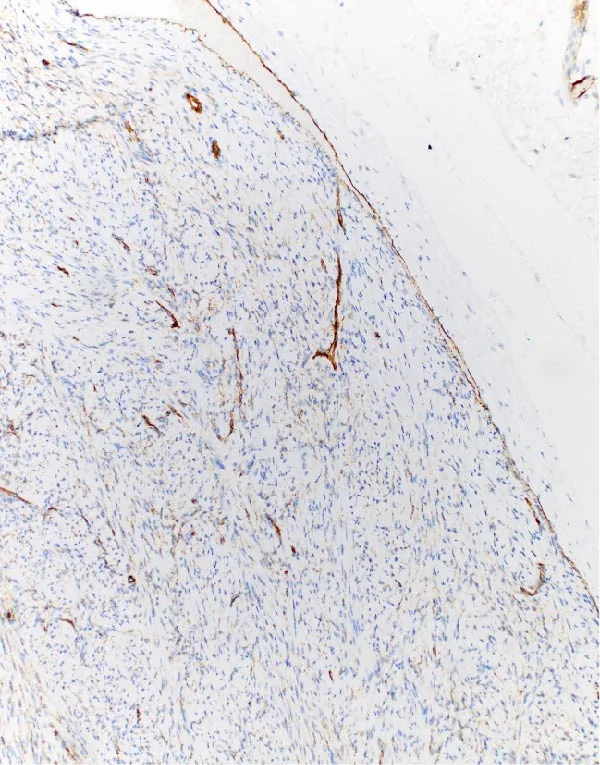
CD31 immunohistochemically stained tissue section (10x).

**Figure 6:**
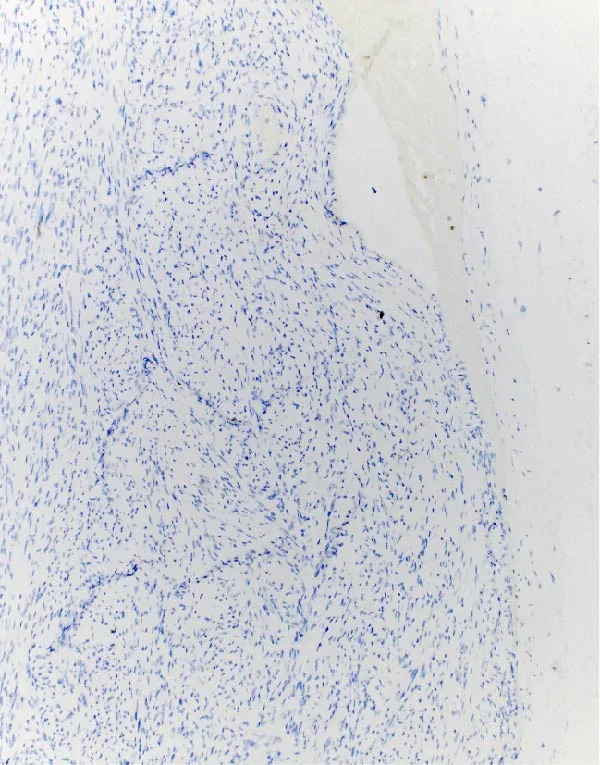
EMA immunohistochemically stained tissue section (10x).

Surgical extirpation revealed a solid tumor adherent to the underlying fascia and muscle that was easily removed along surgical planes. Histopathologic findings on hematoxylin and eosin (H&E)‐stained tissue sections showed a well‐circumscribed, spindle cell lesion with foci of hypercellular areas alternating with hypocellular areas entirely within the circumference of an expanded vein, with a very focal lymphoid cuff (Figures 2A,B). The spindle cells were strongly S100 protein‐positive (Figure 3). An elastic‐stained tissue section marked the single internal elastic lamina around the entire circumference of the blood vessel, supporting that this lesion is a schwannoma entirely within a vein (Figure 4). A CD31 immunohistochemically stained tissue section marked the endothelial cells lining the expanded vein (Figure 5). In addition, a negative EMA immunohistochemically stained tissue section showed a lack of positivity for any perineurial cells, suggesting that this schwannoma did not arise from the vasculature associated with the stroma of a nerve (Figure 6).

The patient has been lost to follow‐up, so any recurrence, complications, or residual deficits cannot be reported at this time. However, it is not expected that surgical intervention would lead to a deficit in functionality.

## 3. Discussion

The case presented shows a benign spindle cell proliferation with Verocay bodies and S100 protein positivity, confirming the diagnosis of schwannoma (Figures 2A,B and 3, respectively). Notably, this temporoparietal scalp mass is entirely intravenous, as demonstrated on a CD31 immunohistochemically stained tissue section and the Verhoeff van Gieson elastic tissue‐stained tissue section, representing a unique presentation (Figures 4 and 5). While schwannomas are typically associated with peripheral nerves, intravascular localization is highly unusual and has only been reported in a limited number of cases. The absence of EMA staining and lack of an associated nerve bundle further support that this lesion did not arise from a conventional intraneural origin or the blood vessel walls found in the stroma of a peripheral nerve but instead most probably represents a true intravenous variant (Figure 6).

As previously mentioned, only four prior cases of intravascular schwannomas have been described in the literature. Reported cases involved the extremities, particularly the posterior calf, or central vascular structures, with lesion sizes ranging from ~0.3 to 1.5 cm. In contrast, the present case demonstrates several distinguishing features, including its location in the temporoparietal scalp, a region not previously reported, and its substantially larger size of 5 × 7 cm^2^. Comparisons are summarized in Table 1. Additionally, unlike previously reported cases that were often incidental or minimally symptomatic, this lesion was clinically suspected to be a cutaneous inclusion cyst in the deep dermis and/or subcutis, highlighting the potential for misdiagnosis and the novelty and relevance of this case report.

**Table 1:** Summary of reported intravascular schwannoma cases in the literature (updated from Dai and Cai).

Reference	Gender	Vascular system	Location	Tumor diameter (cm)	Symptoms	Clinical dx (Pre‐op)	Treatment	Outcome	Key features
Gaudi et al. [6]	56/F	Venous	Right posterior calf	0.3	Mild tenderness	Subcutaneous nodule	Excision	Resolved	First reported intravascular schwannoma
Park and Lee [7]	44/F	Arterial	Middle cerebral artery	1.5	SAH, neurologic deficits	Dissecting aneurysm	Surgical resection + vascular repair	Complicated (post‐op occlusion)	First intra‐arterial case
Ward et al. [8]	54/M	Arterial	Posterior neck	1.2	Painful mass	Not specified	Excision	Resolved	Head/neck location (non‐extremity)
Dai and Cai [1]	47/M	Venous	Right posterior calf	0.8	Nontender enlarging nodule	Subcutaneous mass	Excision	Resolved	Fourth reported case, second venous occurrence
This case	69/M	Venous	Postauricular/temporoparietal scalp	7 × 5	Painful mass	Epidermal inclusion cyst	Excision	Lost to follow‐up	Largest, scalp location, mimicked EIC

The differential diagnosis for an intravascular spindle cell lesion includes a variety of entities, each with a distinct histopathology and immunohistochemical profile. Angioleiomyomas are well‐circumscribed proliferations of benign smooth muscle cells arranged around prominent blood vessels, and they are rarely found in the head and neck region [9, 10]. They also typically demonstrate positivity for smooth muscle actin (SMA) and desmin [11]. Unlike schwannomas, angioleiomyomas lack S100 positivity and do not exhibit Verocay bodies, helping to distinguish them from the presenting case. PEComas are characterized by perivascular epithelioid cells with melanocytic and myoid differentiation and are typically positive for HMB‐45 or Melan‐A rather than S100 [12]. They also usually grow around rather than within vascular lumens, distinguishing them from the intraluminal growth pattern observed in this case [12]. Glomus tumors, derived from modified smooth muscle cells, may rarely exhibit intravascular growth but are SMA‐positive and S100‐negative, distinguishing them from schwannomas [13–15]. Finally, leiomyosarcomas of vascular origin represent an important malignant consideration and typically demonstrate cytologic atypia, mitotic activity, and positivity for SMA and desmin. The absence of these features, along with strong S100 positivity and lack of infiltrative growth, argues against this diagnosis in the presented case [16, 17]. Due to the exceptionally rare occurrence and reporting of intravascular schwannomas, their pathogenesis remains incompletely understood [1]. Proposed mechanisms include origin from small nerve fibers within vessel walls, direct tumor extension from an adjacent schwannoma, or less likely, tumor embolization [6–8]. In this case, origin from nerve fibers within the wall of a vein is favored as exhaustive tissue sections for complete histologic evaluation of the vein do not show extension from a nearby intraneural schwannoma into the vein. No associated nerve was found. It is highly unlikely that this intravenous schwannoma originated from an embolus. The Verhoeff van Gieson elastic tissue‐stained tissue section showed a single internal elastic lamina to the vessel surrounding the schwannoma as well as a faint contiguous rim along the very outer cells of the schwannoma separated from the vein by a cleft in some areas, suggesting that the schwannoma arose from the vein and not from an implanted embolus (Figure 4). The CD31‐marking endothelial cells show the same pattern (Figure 5).

From a clinical perspective, this case underscores several important considerations. First, intravascular schwannoma should be included in the differential diagnosis of slow‐growing subcutaneous masses, particularly when lesions are atypical in location or symptomatology. Second, preoperative diagnosis is often not possible as these lesions may mimic more common entities such as EIC, and definitive diagnosis relies on histopathologic evaluation. Finally, complete surgical excision with clear margins remains both diagnostic and curative, with a seemingly low risk of recurrence. It is likely that the tumor would continue to grow without this treatment.

## Funding

The authors have nothing to report.

## Disclosure

This study was previously presented as an abstract/poster at the 2024 61st American Society of Dermatopathology meeting (November 4–10, 2024, Chicago, Illinois).

## Consent

The authors obtained written consent from the patient for their photographs and medical information to be published in print and online, with the understanding that this information may be publicly available.

## Conflicts of Interest

The authors declare no conflicts of interest.

## Data Availability

The data that support the findings of this study are available upon request from the corresponding author. The data are not publicly available due to privacy or ethical restrictions.
